# Future scenarios of palliative care in health system of Iran: a multi-method study

**DOI:** 10.3389/fpubh.2024.1346234

**Published:** 2024-08-27

**Authors:** Salman Barasteh, Akram Parandeh, Maryam Rassouli, Rohallah Zaboli, Amir Vahedian Azimi, Morteza Khaghanizadeh

**Affiliations:** ^1^Nursing Care Research Center, Clinical Sciences Institute, Baqiyatallah University of Medical Sciences, Tehran, Iran; ^2^Nursing Faculty, Baqiyatallah University of Medical Sciences, Tehran, Iran; ^3^Cancer Research Center, Shahid Beheshti University of Medical Sciences, Tehran, Iran; ^4^Department of Health Administration, Health School, Baqiyatallah University of Medical Sciences, Tehran, Iran; ^5^Nursing Care Research Center, Clinical Sciences Institute, Nursing Faculty, Baqiyatallah University of Medical Sciences, Tehran, Iran; ^6^Behavioral Sciences Research Center, Life Style Institute, Nursing Faculty, Baqiyatallah University of Medical Sciences, Tehran, Iran

**Keywords:** palliative care, end of life care, hospice, uncertainty, drivers, scenario planning, future scenarios, health system of Iran

## Abstract

**Background:**

Paying attention to palliative care has accelerated in Iran in the last 10 years. Considering the trend of aging, increasing burden of chronic diseases and increasing health costs, planning and development of palliative care is necessary in the future. This study was conducted with the aim of explaining the alternative scenarios of palliative care in the health system of Iran until the horizon of 2030.

**Methods:**

This study was a multi-method scenario planning with a qualitative using multiple methods design, which was conducted in 3 phases in 2018–2020. In the first phase, a list of driving forces was extracted using qualitative interviews and literature review. In the second phase, all factors identified in the previous phase were examined in terms of degree of uncertainty and cross-impact analysis, and two key uncertainties were extracted. In the third phase, based on two key uncertainties, four future scenarios of palliative care were formulated, validated and scenario strategies were presented.

**Results:**

The results indicate two uncertainties, including “governance of palliative care in the health system” and “acceptance of palliative care by society,” based on which, four scenarios with the names “climbing to the top,” “excruciating climb,” “edge of the abyss” and “The bottom of the valley” were compiled.

**Conclusion:**

The development of palliative care in health system of Iran is faced with serious uncertainties that it is necessary to focus the developmental activities of palliative care on the two axes of acceptance by society and need for coherent governance by considering all the dimensions and influential components by ministry of health. The application of the results of this research can provide reasonable options for effective interventions and implementation of this category of services to the beneficiaries of palliative care.

## Introduction

The World Health Organization (WHO) defines palliative care (PC) as an approach that improves the quality of life of patients and their families in the face of life-threatening problems, through the prevention and relief of pain and suffering, with rapid diagnosis and evaluation and treatment of pain and other physical, mental and spiritual problems (1). The concept of PC was included in the definition of universal health coverage in 2012 (2). In 2014, the PC resolution at the World Health Assembly emphasized the need for continuous care, at all levels, with emphasis on primary care, community and home care, and universal coverage programs (3). This year, WHO asked its members to formulate policies to ensure the integration of evidence-based, effective and equitable PC in national health services as an element of continuous care (4). PC is one of the main components of a comprehensive response to non-communicable diseases (NCDs) and should be included in the chronic care of all operational plans of countries. However, in 2019, only 50 % of the world’s countries reported PC policies in NCD action plans (5).

Iran as a country that located in the eastern Mediterranean region (EMR) with a low-middle income economic status will face two major challenges of increase in the older adult population and the burden of chronic diseases (6). Also it is predicted that the population of 65 years and above will reach 8,849 per hundred thousand people in 2030 from 5,272 in 2019 (7). Also, mortality from NCDs has increased by 14.5% in the last 20 years (8). This issue will increase the costs imposed on the health system and patients’ families, as well as dissatisfaction and burnout of health system employees. These demographic and epidemiological changes require health system of Iran to adopt with these changes, one of which is the expansion of PC services on a large scale in the country (1).

In the last 10 years, PC has received more attention in EMR (9). The 2015 Quality of Death Index examined PC worldwide and ranked Iran as a country that located in EMR at the bottom of this scale (10). In 2020, the Global Atlas of PC classified Iran in A3 group (11).

In 2013, Iran’s Ministry of health (MOH), in line with its development plan (12), developed a comprehensive national program of palliative and supportive care for cancer in the country (13). Also with the establishment of the nursing deputy in MOH in 2013, this deputy developed counseling centers, nursing services at home, the regulations for long-term care centers and hospices and long-term care centers (14) and planned a pilot plan for the establishment of PC for cancer patients for 4 volunteer centers, and compiled home care service packages for insurance coverage (14).

However, PC services are still in their infancy and are available only in a limited center in major cities. In Iran, most people and their families prefer to receive health services at home, but near death, they tend to receive these services in hospitals (15). The expansion of PC in Iran is faced with challenges such as weak coherent governance, weak infrastructure, weak awareness of society, and poor supply of narcotic drugs (1).

The achievements of these national programs were the very limited establishment of PC in health system of Iran, which has put many challenges and uncertainties in front of decision makers in this field. The lack of success in the National PC Program, has forced planners and policy makers to select plan scenarios and consider alternative futures. By limiting future uncertainties to a limited number of drivers and influencing factors, scenarios present simpler futures to policy makers, decision makers and stakeholders (14). Strategic decision-making processes and forward-looking planning are also important issues that are mainly discussed by the stakeholders of the health system (16). However, the world of the future is full of changes and instability, and only those who can actively move toward them can endure these changes. The pace of change is so fast that traditional methods cannot keep up with it. Therefore, countries must be forward-looking to deal with driving changes that affect all aspects of their lives. Public health foresight and, accordingly health and science foresight are essential in promoting health and solving related problems (17).

Foresight science has facilitated future studies and turned future-oriented planning studies into science with precise principles and methods. The process of foresight helps to identify and introduce driving forces, analyze their direct and indirect effects in a dynamic system and determine the critical variables of planning for the future (18) and finally provides the necessary facilities for policy makers in a strategic and long-term context (19). It seems that providing a centralized action plan will not be a suitable solution for formulating and drawing the future of PC in health system of Iran. Therefore, the present study was conducted to explore future scenarios of palliative care in health system of Iran.

## Methods

**Figure 1:**
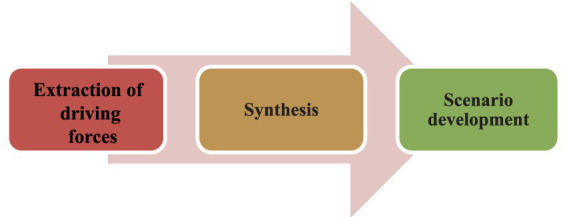
Phases of scenario planning.

This was a scenario planning study that approved by the Ethics Committee of Baqiyatullah University of Medical Sciences (AS) with code IR.BMSU.REC.1397.021. The design of the study was qualitative using multi-method, which was conducted in 3 phases from 2018 to 2020. To conduct this study, the steps of the deductive scenario analysis described by Van der Heijden were used (19). In the first phase, the external environment was examined to extract driving forces using literature review and qualitative interviews with policy makers. Then, in the second phase, the factors identified in the previous phase were ranked in terms of degree of uncertainty and impact rate, and two key uncertainties were explained. Finally, in the third phase, four future scenarios of palliative care were developed and validated based on two key uncertainties according to experts’ opinion (Figure 1).

### Phase Ι: exploring the extraction of driving forces

In this phase, a list of influential driving forces was extracted using qualitative interviews and literature review.

#### Step 1—interview

Semi-structured interviews were conducted with 17 palliative care (PC) policy makers, decision makers, patient and their family caregivers based on purposive sampling (demographic data presented in Table 1). The interviews ended after reaching data saturation. The time of the interviews was 17 to 60 min. To conduct the interview, the researcher first introduced himself, the title, general and partial objectives, and the possible time of the interview to the participants. Then permission was obtained to record the interviews and refer again to the participants. The main question “How do you see the current and future challenges of PC in health system Iran?” was asked. Then, exploratory questions such as “Since the concept of PC was introduced in the country until now, what changes have you witnessed?” or “What needs to happen if the PC system is to be fully deployed?” according to the experiences expressed by the participant, was asked. At the end, the participants were told that the researcher’s questions and topics were finished, if you have any other points or questions, please mention them. Immediately after conducting each interview, the interviews were written down and the interviews were reviewed several times to gain an understanding of the entire interview. The analysis of the handwritten interviews was done based on the five-step method of Framework analysis (18). The extracted codes were classified during the reduction and condensation process using MAXQDATA 10 software (20). Credibility was done through two methods; member check and peer check. For member check, the interviews were read several times by the research team and checked with the participants after coding the interviews. Also they were asked to provide corrective comments about the codes. For peer check, the data was independently coded and categorized by the first author, and the extracted categorizes were analyzed. Then it was sent to the research team. In cases of disagreement, the discussion between the research team continued until reaching an agreement. When the authors disagreed, discussions and clarifications continued until agreement was reached. The study report presented using the consolidated criteria for qualitative reporting research (COREQ) (21).

**Table 1:** Demographic information of interviewees.

Number	Gender	Experience (year)	Interview duration (minute)	Position
1	Female	10	25	Faculty member/researcher
2	Female	10	45	Vice-Chancellor of Charity Education/Physician
3	Female	7	40	General practitioner
4	Male	4	20	Charity manager
5	Male	14	50	Oncologist
6	Male	10	22	Specialist in Pediatric Oncology/Chairman of Charity Board
7	Male	8	15	Pain specialist
8	Female	10	26	Obstetrics specialist
9	Male	7	30	Faculty member/researcher
10	Female	6	60	Faculty member/researcher
11	Female	-	25	Patient caregiver
12	Female	-	30	Patient caregiver
13	Female	-	40	Patient
14	Female	-	45	Patient
15	Male	5	35	Clergyman/spiritual caregiver
16	Male	3	20	Faculty member/researcher
17	Male	5	35	Psychologist

#### Step 2—literature review

The purpose of this step was to conduct a comprehensive literature review on factors affecting the future of PC. To access the international documents, the databases including PubMed, ISI Web of Science, Scopus, EMBASE and WHO website were searched. Also Farsi databases including Magiran, Scientific Information Database (SID), as well as the websites of the MoH, the Islamic Council, and universities of medical sciences were searched. The combination of Farsi and English keywords including; trends, “foresight of palliative care,” “scenario of palliative care,” “palliative care,” “end of life care,” hospice, “terminal care,” social, technological, economic, environmental, political were searched without time limitation. The inclusion and exclusion criteria of the text were determined based on the following three items; include social, economic, political, technological and ecological trends; are related to PC and published between 2000 and 2018 in Farsi and English. The extracted drivers were analyzed based on the STEEP model. The STEEP model is one of the most common methods of examining the external environment, which includes the first letters of the words social, technological, economic, environmental and political (22).

### Phase II: synthesis

**Figure 2:**
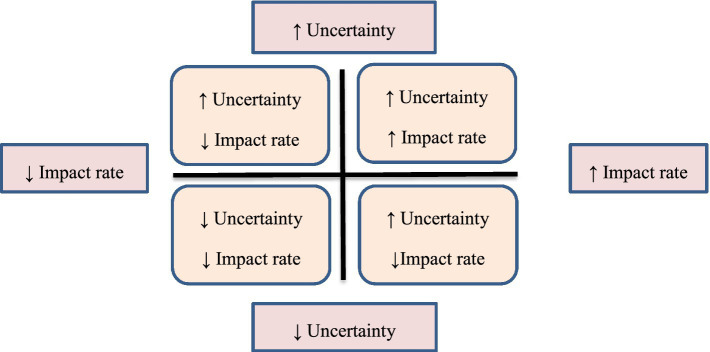
Scenario matrix.

In this phase, all identified factors in the exploration phase were ranked in terms of uncertainty and impact rate. For this purpose, a questionnaire including influential factors was sent to 17 experts and they were asked to prioritize on a scale of 1 (low/weak) to 10 (high/strong). Then the results were drawn on a 2*2 matrix as the impact/uncertainty matrix. The X axis was uncertainty and the Y axis was uncertainty. In this matrix, the factors with the highest uncertainty and the highest degree of impact were identified as critical uncertainty in the upper right quadrant and were identified based on similarity in two categories of key uncertainty (Figure 2). These two extracted key uncertainties are the basis of matrix formulation.

### Phase III: scenario development

**Figure 3:**
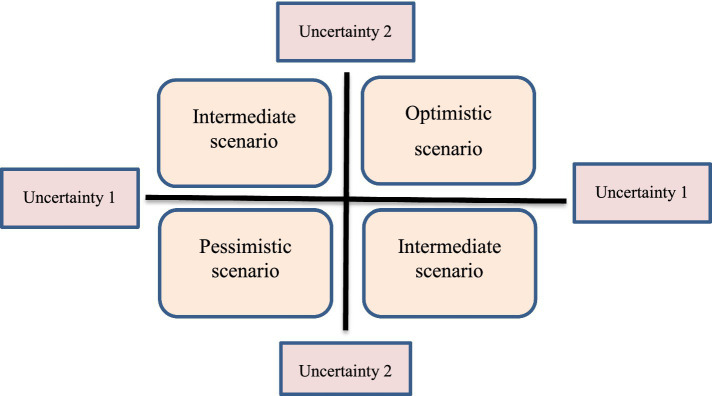
The matrix of scenarios based on two key uncertainties.

In this phase, based on two key uncertainties, four future scenarios of PC were creatively developed and validated. In the evaluation of the validity of the scenario, the quality and characteristics of the scenarios are evaluated in relation to the goal. The scenarios developer may return them to the participants involved in the scenario developing process and ask them to review the scenarios. But sometimes it can also use people outside the scenario developing process. A simple survey aimed at identifying the main characteristics of a good scenario is suggested by Wiseman et al. as follows: decision-making power, plausibility, range of alternatives, differentiation, logical consistency, memorability, challenging to perception about future (23). These two key uncertainties were placed on the X and Y axis in two very negative and very positive states and create four quadrants (Figure 3).

## Results

### Phase I: extraction of driving forces

13 drivers were extracted from qualitative interview, 12 drivers from analysis of national documents (21), and 24 drivers from international documents.

### Phase II: ranking of synthesis forces and explanation of two key uncertainties

The drivers extracted in the qualitative interview and review of national and international documents were placed in the form of the STEEP model (24). STEEP (Societal, Technological, Economical, Environmental, and Political) analysis looks at the relevant parts of the global trends for manufacturing and applies proven methodologies to evaluate the importance of technology research topics for scenario developed from a subset of the global trends (25). After preparing the table, it was made available to the experts. According to the opinions of the experts, 12 drivers got the highest impact rate and uncertainty score (Table 2). Based on the similarity, two key uncertainties named “governance of PC in the health system” and “Acceptance of PC by society” were extracted from the drivers.

**Table 2:** Drivers and uncertainties shaping the scenarios.

Drivers	Uncertainties
Changing the thought patterns of decision-makers regarding PC	Governance of palliative care in the health system
Improving inter-cooperation and coherence in policies	
Sanctions and unilateral pressures	
Inclusion of PC in the basic package of health services	
Integration of PC into the health system	
Expansion of outpatient services and long-term care	
Establishing PC centers such as inpatient departments and hospices	
Expansion of PC with the help of telehealth and health information technology	
Expansion and promotion of home care services	
Improving the knowledge and attitude of society toward caring for the dying person	Acceptance of palliative services by the community
Expanding health literacy and public health knowledge	
Increasing people’s expectations by expanding knowledge and communication	

### Phase III: scenario development

**Figure 4:**
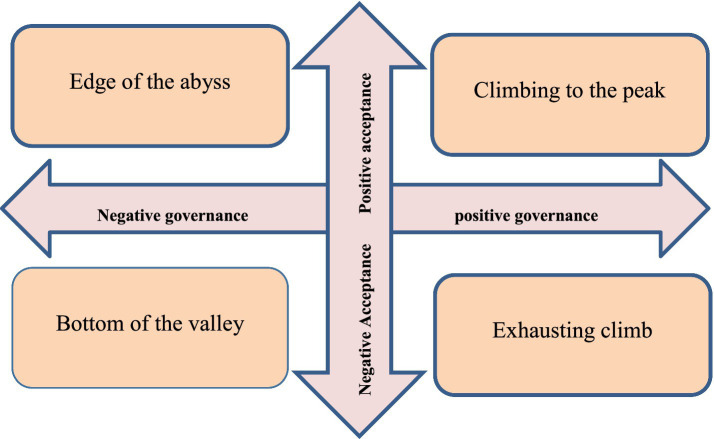
Two-by-two matrix related to two key uncertainties and formation of four scenarios.

Based on these two uncertainties, four scenarios named “climbing to the peak,” “exhausting climb,” “edge of the abyss” and “bottom of the valley” were creatively developed (Figure 4). The summary of the scenarios is presented in Table 3. Also, the results of evaluating the validity of the scenarios using the opinions of 12 experts showed that the scenario of climbing to the peak gained the most credibility. Therefore, it is suggested that this scenario should be taken into consideration by policy makers and officials as reasonable options.

**Table 3:** Summary of scenarios at glance.

Scenario	Bottom of the valley	Edge of the abyss	Exhausting climb	Climbing to the peak
Drivers				
Changing the thought patterns of decision-makers	Unchanged	A little changed	Somewhat changed	Excellent
Coherent policy making	Weak	A little	Good	Excellent
Sanctions	Not resolved at all	Not resolved at all	Somewhat resolved	Resolved
Inclusion of PC in the basic package of health services	Not at all	Not at all	Not at all	Completely
Integration into the health system	Undesirable	A little	A little	Desirable
Outpatient services and long-term care	Weak	A little	Somewhat	Excellent
Inpatient departments and hospice	Not at all	A little	Somewhat	A lot
Telehealth and health information technology	Not at all	A little	Somewhat	A lot
Home care services	Weak	A little	Medium	Desirable
Knowledge and attitude of community	Weak	A little	Good	Desirable
Health literacy and public health knowledge	Weak	A little	Medium	Desirable
People’s expectations	Weak	A little	Somewhat	Completely

#### Scenario 1: climb to the top

This scenario describes the most optimistic scenario as a PC utopia. In this scenario, PC has a specific Trustee. The activities are coherent and supporting laws have been passed in the parliament and MOH. Palliative services have a tariff. Basic health care package is included and insurances cover these services. There are three levels of health services, providing and referring patients from level one to level three. In remote areas, these services are provided using tele-palliative and mobile palliative technologies. Care centers such as clinics, care centers and hospices and home care provide a wide range of services. The awareness of society (policy makers, providers and people) is at a very high level. The process of demanding and supplying to society has been formed. The media is seriously educating the society; In a way that society has become aware of their rights. In this scenario, due to the high mortality rate of COVID-19, the need to provide palliative services, especially psychological and spiritual services, is very tangible. In this regard, the general managers of the health system are rapidly expanding palliative services and using the experiences gained from this pandemic in the development of palliative services.

#### Scenario 2: exhausting climb

In this scenario, the nursing deputy of MOH is the trustee of PC. The nursing deputy has effective cooperation with other deputy of MOH, insurance organization, and charity organization. However, PC laws have not yet been legislated. The use of communication technologies is not enough to reduce the burden of referrals to the emergency department of hospitals. Palliative services are not yet included in the basic package. The services are covered by some insurances. Initial plans for the integration of PC in the PHC and referral system between the deputy of nursing, the health deputy, and the treatment deputy have been made, but they have not been implemented. The regulations for the establishment of care centers have been announced by the Ministry of Health’s Deputy Director of Treatment, and centers in big cities have announced their readiness. Home care services have been activated by private centers. Society’s awareness, society’s persuasion to receive services from therapists and society’s demands have increased.

However, the distribution of services in the country is not enough and access to these services is limited in rural areas and small towns. The expansion of health and public health literacy related to PC and the community’s interest in receiving these services at the community level, the search and demand of people about the preferred place for end-of-life care and death caused the officials to think about the expansion of these services at the community level. On the other hand, due to the widespread spread of COVID-19, it seems that reaching the desired situation is facing challenges. A large amount of the budget of the health system is allocated to the treatment of the disease. People in the society have faced stigma and many psychological risks. Therefore, it is necessary to expand palliative services for patients and their families.

#### Scenario 3: the edge of the abyss

In this exploratory scenario, due to the spread of the pandemic and the serious effects of the pandemic on the health system, the country has faced a serious economic and social crisis. Incoherent activities have been carried out to relieve the pain and provide palliative services to the patients and their families, but it is not considered as the next service package provided to the patients. The health system is unable to provide routine services to the patients and adequate palliative services are not provided to the patients. However, few centers and sometimes charity centers provide these services to patients sporadically.

#### Scenario 4: the bottom of the valley

This exploratory scenario represents the most pessimistic possible scenario. In this scenario, policy makers have not yet realized the importance of PC. Supporting laws has not been legislated. Providers and receivers are not aware of the benefits of technologies. Visits of PC specialists are done in person. The remote areas do not have any access to services. PC is not integrated into the basic service package and there is no insurance coverage for the service. Only people with high financial status have access to services. Services are provided sporadically by charity centers. Patients in need of PC still visit emergency department of hospitals frequently. Outpatient service centers, long-term centers, hospital wards, and hospices have not yet been activated. Home care in big cities is limited to acute services. Awareness and knowledge of PC is limited to researchers, and there is no adequate information on PC and life-threatening illness. People are mostly interested in treatment rather than care. Health literacy related to end-of-life decision-making is at a disadvantage. In such a way, expensive treatments and numerous surgical procedures are still performed for people who need PC. People are poorly aware of their rights in the face of life-threatening illness and expect people to be treated until the moment of death. The demand-response process has not been formed. The spread of the COVID-19 pandemic was the main surprise that overshadowed the entire forecasting process. In the “Bottom of the Valley” scenario, a large part of the health budget will be spent on dealing with this pandemic, and little attention will be paid to the expansion of PC in the country.

### The wildcard of COVID-19

Wildcards are a group of events that have a very low probability of occurrence; but with their occurrence, the future will be greatly affected. The outbreak of the COVID-19 quickly affected all countries of the world. In such a way that the WHO introduced it as a global pandemic in March 2020. The pandemic is the most important health issue in Iran and all countries of the world. The outbreak of this pandemic had a serious impact on the current foresight projects. With the spread of this pandemic, a large part of the health service budgets was allocated to the control of this disease. An unprecedented economic recession has happened all over the world and in Iran. In addition, Iran was also facing the economic sanctions of the United States. The effects of these two challenges had serious effects on the health of the Iranian society. It seems that these two challenges of health system of Iran will face more serious crises in the future. It will also cause difficulties in allocating funds and passing laws in the parliament to support the provision of palliative services. The existence of concepts such as physical, mental, and spiritual care, care at the preferred place of the patient and his family, as well as the low costs of providing these services can raise the need to provide them on a large scale in the global pandemic conditions.

## Discussion

The present study was conducted to develop the alternative scenarios of PC in health system of Iran until the horizon of 2030. The reason for choosing Horizon 2030 is to achieve sustainable development of health in the field of palliative care, which is mainly considered by various countries committed and non-committed to this international document for their planning. The results of this study were four alternative scenarios including “climbing to the peak,” “exhausting climbing,” “edge of the abyss” and “bottom of the valley.” Based on the opinions of the experts, it seems that the “climbing to the top” scenario is more likely to occur. In these scenarios, drivers have been considered for the development of scenarios that have different development in various scenarios. These drivers including incoherent governance, integration in the referral system and primary health care, technology, inclusion to the basic health package, creating care centers including hospice, clinic, home care, public awareness and health care providers’ awareness.

In Iran, as one of the countries of EMR, palliative care is inaccessible to most people in need. Overall, EMR has significantly fewer palliative care services than the other five WHO regions. No country in the region has achieved the integration of palliative care into MoH (26). So far, no such study has focused on the development of future scenarios of palliative care in the health system of different countries. Therefore, it is not possible to compare palliative care scenarios in the present study with other future scenarios in other countries. Hence, we focused on the drivers or indicators of shaping the future of palliative care.

According to the results of the present study, Khanali Majen has also mentioned the following directions for the development of palliative care in the future of Iran. These directions included; establishing a variety of palliative care centers, providing access to palliative care for all patients, preparation of standard care services package, development of an independent discipline, providing palliative care courses as part of the curriculum, and establishing international links for education and research (27). Also WHO introduced palliative care development indicators for countries in 2021. Many of these indicators are similar to our extracted drivers. These indicators includes specialized palliative care programs for adults and children, existence of a national palliative care plan, inclusion of palliative care in the list of health services provided at the primary care, existence of national coordinating authority for palliative care, report and availability of essential medicines and opioid, proportion of medical and nursing schools with palliative care formal education, palliative care research, existence of groups dedicated to promote the rights of patients, their caregivers, and disease survivors, existence of national policy for advance care planning (28).

### Guideline recommendations for moving the PC toward better future

The first pillar of future of PC is coherent policy and governance. WHO has mentioned coherent policy making as one of the key pillars of PC development (28, 29). Having national policies leads to increased access to PC services, and successful and leading countries in PC services have a comprehensive and targeted policy framework, and PC is integrated into their health system (30). However, PC policy is done in different ways in various countries. In some countries, PC is a stand-alone program or a part of national health programs, and in others, it is a component of the cancer control program (31). In Iran, PC policy making is one of the serious challenges and several studies have addressed this issue (32). Therefore, it seems that coherent governance will be one of the essential pillars of the future scenarios of PC.

Providing integrated services at different levels of the health system is another indicator of the future of PC. WHO has also acknowledged the integration of PC at different levels of service delivery in several reports (28, 29) and specifically the integration in PHC (33). The four countries of New Zealand, Canada, Australia, and England have different structures for providing services in a leveled manner in general services and specialized services (34). Ignoring the leveling and referral system causes abandonment of treatment, confusion of patients, unnecessary referrals and waste of resources (32). In Australia, PC services are provided in two ways: public and private, where public services are available everywhere and specialized services are divided into three levels based on the population of the regions (35). PC in Italy is in the form of a regular care network that reduces admission to the emergency room and length of hospital stay and increases satisfaction (36). Also, several studies in the world have supported the integration of PC in PHC and several benefits have been reported. Alshammary et al.’s study pointed to improved access to primary PC, improved symptom control, adaptation to cancer treatment, improved quality of life, and overall satisfaction (37). Bakitas et al. have also mentioned improving the quality of life of patients and caregivers in advanced cancer care (38). In Iran, based on the model proposed by Heydari et al. (39), incurable patients are identified and health care is provided reversely from specialized centers to patients’ homes. Also, the analysis of Iran’s upstream documents has explained the “requirements of integrating PC into primary health care” including the principles and bases, legislation and policy making, the establishment of the PC system and civil support. Therefore, it is recommended that the principles and foundations of this care be explained in the country and then the necessary infrastructure for this integration be provided with the cooperation of government organizations, NGOs and charities with appropriate policies.

In order to include palliative services in UHC and providing insurance services, insurance organizations face many challenges, including economic challenges due to the multitude of insurance organizations, the overlap of the insured, and the lack of use of economic evaluations to cover service (40). The existence of home care and end-of-life care service packages in Canada and England has provided easy access to services at no cost. A service package is developed with the aim of increasing people’s access to health services and financial support for people (41). Policymakers and insurers should identify cost-effective services and provide adequate access to national packages. Obviously, without a clear definition of the tariff, it is not possible to provide these services.

One of the most important places of death is PC centers. These care centers provide translational services as a link between home and hospital. The most important desert centers include hospice, nursing homes, long term care facilities (LTCFs), consult clinics. In the last 10 years, European countries have greatly increased the number of care home centers in order to access health services (42). In recent years, there have been significant changes in the PC system in the form of a change in the place of care from hospital to home. Home care is one of the most desirable models of providing PC (43). Home-based PC has very beneficial effects on the physical, spiritual, psychological, social and economic aspects of patients and reduces the costs of the health system, shortens the length of hospitalization, reduces complications, continues care and prevents re-hospitalization of patients (44, 45). Also, the WHO has introduced home-based PC as one of the main elements of health systems in 2014 (46). Considering the increase in the older people and the carelessness of these people and the lack of need for acute hospital care, the necessity of such centers in Iran is serious.

The next indicator is telepalliative care and telemedicine. By using remote PC, services are extended equitably to the deprived and remote areas. According to Jabari et al.’s study, PC is not provided in rural and remote areas; but it can be facilitated by using Family physician plan and with the help of computer systems (47). The Pinto study reported similar benefits for telehealth in PC as other health studies (48). Therefore, the implementation of telehealth appears to be a promising approach to address some of the challenges in providing and distributing PC (49). By providing health services to hospitalized people at home who cannot move, medical services can be transferred from the hospital or clinic to the home using communication technology (50). Therefore, the application of telemedicine is a better way to provide equitable PC.

Community acceptance of PC includes community and PC provider awareness and the role of media. Several studies have identified the lack of awareness and knowledge as a major barrier to the expansion of PC (51, 52). People in society still deny death. Talking about death and dying is still considered taboo (53, 54). This misconception of patients and their families about the concept of PC has been reported as a serious obstacle for public education (55). In line with the present study, McIlfatrick et al. have mentioned that it is essential to modify people’s attitudes toward PC in order to achieve care goals and greater community participation in PC and end of life (55). However, Cox et al. have noted that public attitudes regarding PC and end-of-life care are complex and ambiguous (56). Therefore, it is possible to change the society’s attitude with authentic educations that are appropriate to social trends, use of advertising posters, free discussions at the community level by health service providers in order to raise the awareness of the society.

In the current study, the media is an important indicator of the future of PC. From the point of view of medical sociology, how to examine health-related issues in the media is important (57). Media in the construction of identity and experience in the field of health is examined by medical sociologists (58). Van den Berg et al.’s study mentions that the influence of these media should not be underestimated when building the social image of PC (59). On the other hand, numerous studies have mentioned that modifying public perceptions of PC is the basis of improving knowledge and access to services, empowering people, and community participation in PC and end-of-life care. Efforts to improve public awareness of PC are possible by considering cultural, demographic (56, 60–62), ethnographic (63) and social structure characteristics, including dependency (61, 64).

In a systematic study, Li et al. (65) and Parajuli et al. (66) showed that nurses’ mastery of PC knowledge is not enough. Khanali-Mojen et al. also showed that Iranian doctors and nurses have average knowledge and attitude toward PC (67). Therefore, it seems that the knowledge of PC providers in Iran, like in other countries, can be one of the serious challenges for the development of PC in the future.

## Conclusion

Scenario planning provides various conditions of the future situation to the stakeholders. Using the scenarios depicted in the present study, the future needs of Iran’s PC will be explained and made available to managers, policy makers and beneficiaries of PC. In order to reach the optimal state, it is necessary to develop the quality and quantity of PC by establishing a clear responsibility, developing supporting laws, pricing and including the basic health package, insurance coverage of services, integration in the three levels of health services using modern and developing technologies. Care centers should be done by increasing the awareness of society (policy makers, providers and people).

### Limitation

Due to the multi-method nature of the study, first a qualitative study was conducted with experts. The qualitative study had limitations such as difficult access to policy makers. Also, developing scenarios had challenges. First, the experts were not familiar with the process of developing the scenario. For this challenge, we first held a workshop for them and taught them the process of scenario developing.

### Future studies

After developing the future scenarios of PC, it is suggested that each of the desirable future indicators of PC include governance, integration in the referral system and PHC, technology, closed inclusion of the basic health package, care centers including hospice, clinic, home care, public awareness, health literacy and public health should be studied separately. Also operational and specific plans to achieve each indicator should be developed.

## Data Availability

The raw data supporting the conclusions of this article will be made available by the authors, without undue reservation.

## References

[1] Barasteh S Rassouli M Parandeh A Vahedian-Azimi A Zaboli R Khaghanizadeh M. Palliative care in the health system of Iran: a review of the present status and the future challenges. Asian Pac J Cancer Prev. (2020) 21:845–51. doi: 10.31557/APJCP.2020.21.3.845, PMID: 32212816 PMC7437322

[2] Knaul FM Farmer PE Krakauer EL De Lima L Bhadelia A Kwete XJ. Alleviating the access abyss in palliative care and pain relief—an imperative of universal health coverage: the lancet commission report. Lancet. (2018) 391:1391–454. doi: 10.1016/S0140-6736(17)32513-8, PMID: 29032993

[3] Alliance WHPC. Universal health coverage and palliative care. London: Worldwide Hospice Palliative Care Alliance (2014). 28:130–4. doi: 10.3109/15360288.2014.911801

[4] World Health Organization. Strengthening of palliative care as a component of integrated treatment throughout the life course. Journal of Pain & Palliative Care Pharmacotherapy, (2014). 10.3109/15360288.2014.911801 24779434

[5] World Health Organization. Palliative Care for Noncommunicable Diseases World Health Organization (2020).

[6] Hosseini LJ Samadi AH Woldemichael A Gharebelagh MN Rezaei S Rad EH. Household overcrowding in Iran, a low-middle-income country: how major of a public health concern is it? J Prev Med Public Health. (2021) 54:73–80. doi: 10.3961/jpmph.20.568, PMID: 33618502 PMC7939753

[7] United Nations. Department of Economic and Social Affairs, Population Division. World Population Ageing 2019 (ST/ESA/SER.A/444). (2020).

[8] Khorrami Z Rezapour M Etemad K Yarahmadi S Khodakarim S Mahdavi Hezaveh A. The patterns of non-communicable disease multimorbidity in Iran: a multilevel analysis. Sci Rep. (2020) 10:3034. doi: 10.1038/s41598-020-59668-y, PMID: 32080215 PMC7033095

[9] Fereidouni A Rassouli M Kianian T Souri H Elahikhah M Azizi S. Factors related to good death in the eastern Mediterranean region: a systematic review. East Mediterr Health J. (2022) 28:601–9. doi: 10.26719/emhj.22.069, PMID: 36134492

[10] Brant JM Al-Zadjali M Al-Sinawi F Mushani T Maloney-Newton S Berger AM. Palliative care nursing development in the Middle East and Northeast Africa: lessons from Oman. J Cancer Educ. (2021) 36:69–77. doi: 10.1007/s13187-021-02044-9 34129197 PMC8204607

[11] Clark D Centeno C Clelland D Garralda E López-Fidalgo J Downing J. How are palliative care services developing worldwide to address the unmet need for care? Ed. Connor SR. London, UK: Worldwide Hospice Palliative Care Alliance. (2020).

[12] Mahdavi, M Sajadi HS. Qualitative analysis of Iranian sixth five-year economic, social, and cultural development plan from universal health coverage perspective. BMC Health Serv Res. (2021). 21:966. doi: 10.1186/s12913-021-06985-1 34521388 PMC8442454

[13] Barasteh S Parandeh A Rassouli M Zaboli R Vahedian-Azimi A Khaghanizadeh M. Integration of palliative care into the primary health care of Iran: a document analysis. Middle East J Cancer, (2021) 12:292–300. doi: 10.30476/mejc.2020.82856.1111

[14] Barasteh S Rassouli M Karimirad MR Ebadi A. Future challenges of nursing in health system of Iran. Frontiers. Public Health. (2021) 9:160. doi: 10.3389/fpubh.2021.676160 PMC8345111 34368051

[15] Fereidouni A Rassouli M Karami M Pakseresht M Barasteh S. Preferred place of death challenges the allocation of health resources in Iran. Int J Palliat Nurs. (2023) 29:553–4. doi: 10.12968/ijpn.2023.29.11.553, PMID: 38039122

[16] Vollmar HC Ostermann T Redaèlli M. Using the scenario method in the context of health and health care–a scoping review. BMC Med Res Methodol. (2015) 15:1–10. doi: 10.1186/s12874-015-0083-1 26475601 PMC4609149

[17] Haghdoost A Pourhosseini SS Emami M Dehnavieh R Barfeh T Mehrolhassani MH. Foresight in health sciences using CLA method. Med J Islam Repub Iran. (2017) 31:492–9. doi: 10.14196/mjiri.31.84 PMC6014787 29951385

[18] van der Heijden Kees. Scenarios: The Art of Strategic Conversation. 2nd ed. England: John Wiley & Sons. (2005).

[19] Bialek R Beitsch LM Moran JW. Solving population health problems through collaboration. New York: Routledge. (2017).

[20] Polit DF Beck CT. Nursing research: principles and methods. (2004). Lippincott Williams & Wilkins, Philadelphia, PA.

[21] Tong A Sainsbury P Craig J. Consolidated criteria for reporting qualitative research (COREQ): a 32-item checklist for interviews and focus groups. Int J Qual Health Care. (2007) 19:349–57. doi: 10.1093/intqhc/mzm042, PMID: 17872937

[22] Burt G Wright G Bradfield R Cairns G Van Der Heijden K. The role of scenario planning in exploring the environment in view of the limitations of PEST and its derivatives. Int Stud Manag Organ. (2006) 36:50–76. doi: 10.2753/IMO0020-8825360303

[23] Wiseman J Biggs L Rickards Edwards T. Scenarios for climate adaptation: guidebook for practitioners. University of Melbourne, Carlton, Victoria, Australia. (2011).

[24] Akinyele D Belikov J Levron Y. Challenges of microgrids in remote communities: a STEEP model application. Energies. (2018) 11:432. doi: 10.3390/en11020432

[25] Szigeti H Messaadia M Majumdar A Eynard B STEEP analysis as a tool for building technology roadmaps. In: *Internationale challenges e-2011 conference*; (2011).

[26] Krakauer EL Kwete XJ Rassouli M Arreola-Ornelas H Ashrafizadeh H Bhadelia A. Palliative care need in the eastern Mediterranean region and human resource requirements for effective response. PLOS Global Public Health. (2023) 3:e0001980. doi: 10.1371/journal.pgph.0001980, PMID: 37922240 PMC10624269

[27] Mojen LK. Palliative care in Iran: the past, the present and the future. Support Palliat Care Cancer. (2017) 1:929. doi: 10.22037/spc.v1i1.11929

[28] World Health Organization [WHO]. Assessing the development of palliative care worldwide: a set of actionable indicators. (2021). Retrieved from: https://apps.who.int/iris/handle/10665/345532

[29] World Health Organization. Planning and implementing palliative care services: a guide for programme managers. Geneva: World Health Organization (2016).

[30] Murray S. The quality of death index ranking palliative care across the world. De Economist. (2015) Economist Intelligence Unit: The Quality of Death Index: Ranking palliative care across the world.

[31] Alikhani M Vatankhah S Gorji HA Ravaghi H. How cancer supportive and palliative care is developed: comparing the policy-making process in three countries from three continents. Indian J Palliat Care. (2020) 26:72–9. doi: 10.4103/IJPC.IJPC_55_19, PMID: 32132789 PMC7017679

[32] Ansari M Rassouli M Akbari ME Abbaszadeh A Akbarisari A. Palliative care policy analysis in Iran: a conceptual model. Indian J Palliat Care. (2018) 24:51–7. doi: 10.4103/IJPC.IJPC_142_17, PMID: 29440807 PMC5801630

[33] World Health Organization. Integrating palliative care and symptom relief into primary health care: A WHO guide for planners, implementers and managers. Geneva: World Health Organization (2018).

[34] Nasrollahpour Shirvani D Ashrafian Amiri H Motlagh ME Kabir MJ Maleki MR Shabestani Monfared A. Evaluation of the function of referral system in family physician program in Northern provinces of Iran: 2008. J Babol Univ Med Sci. (2010). 11:46–52.

[35] Mojen LK Rassouli M Eshghi P Zendedel K Sari AA Karimooi MH. Pediatric palliative care in Iran: applying regionalization of health care systems. Asian Pac J Cancer Prev. (2018) 19:1303. doi: 10.22034/APJCP.2018.19.5.1303 29802691 PMC6031829

[36] Rusalen F Agosto C Brugnaro L Benini F. Impact of the regional pediatric palliative care network on the Care of Children on long-term ventilation: could the availability of a residential solution into the network reduce the duration of intensive care unit staying for these patients? J Pediatr Intens Care. (2018) 7:075–80. doi: 10.1055/s-0037-1605369 PMC6260342 31073474

[37] Alshammary SA Duraisamy BP Salem L Altamimi A. Integration of palliative care into primary health care: model of care experience. Cureus. (2020) 12:8866. doi: 10.7759/cureus.8866 PMC7386098 32754404

[38] Bakitas M Lyons KD Hegel MT Balan S Brokaw FC Seville J. Effects of a palliative care intervention on clinical outcomes in patients with advanced cancer: the project ENABLE II randomized controlled trial. JAMA. (2009) 302:741–9. doi: 10.1001/jama.2009.1198, PMID: 19690306 PMC3657724

[39] Hojjat-Assari S Rassouli M Madani M Heydari H. Developing an integrated model of community-based palliative care into the primary health care (PHC) for terminally ill cancer patients in Iran. BMC Palliat Care. (2021) 20:1–11. doi: 10.1186/s12904-021-00795-2 34182980 PMC8240381

[40] Davari M Haycox A Walley T. Health care financing in Iran; is privatization a good solution? Iran J Public Health. (2012) 41:14–23. PMID: 23113205 PMC3469022

[41] Teerawattananon Y Tangcharoensathien V. Designing a reproductive health services package in the universal health insurance scheme in Thailand: match and mismatch of need, demand and supply. Health Policy Plan. (2004) 19:i31–9. doi: 10.1093/heapol/czh043 15452013

[42] Eurofound. Care homes for older Europeans: Public, for-profit and non-profit providers. Luxembourg, Europe: Publications Office of the European Union Luxembourg, Europe (2017).

[43] Farahani AS Rassouli M Mojen LK Ansari M Ebadinejad Z Tabatabaee A. The feasibility of home palliative Care for Cancer Patients: the perspective of Iranian nurses. Int J Cancer Manage. (2018) 11:114. doi: 10.5812/ijcm.80114

[44] Gans D Hadler MW Chen X Wu S-H Dimand R Abramson JM. Cost analysis and policy implications of a pediatric palliative care program. J Pain Symptom Manag. (2016) 52:329–35. doi: 10.1016/j.jpainsymman.2016.02.020, PMID: 27233140

[45] Kerr CW Donohue KA Tangeman JC Serehali AM Knodel SM Grant PC. Cost savings and enhanced hospice enrollment with a home-based palliative care program implemented as a hospice–private payer partnership. J Palliat Med. (2014) 17:1328–35. doi: 10.1089/jpm.2014.0184, PMID: 25375799

[46] Heydari H. Home-based palliative care: a missing link to patients’ care in Iran. J Hayat. (2018) 24:97–101.

[47] Jabbari H Azami-Aghdash S Piri R Naghavi-Behzad M Sullman MJ Safiri S. Organizing palliative care in the rural areas of Iran: are family physician-based approaches suitable? J Pain Res. (2019) 12:17–27. doi: 10.2147/JPR.S178103 30588076 PMC6302809

[48] Pinto S Caldeira S Martins JC. E-health in palliative care: review of literature, Google play and app store. Int J Palliat Nurs. (2017) 23:394–401. doi: 10.12968/ijpn.2017.23.8.394, PMID: 28854054

[49] Rogante M Giacomozzi C Grigioni M Kairy D. Telemedicine in palliative care: a review of systematic reviews. Annali dell'Istituto superiore di sanita. (2016) 52:434–42. doi: 10.4415/ANN_16_03_16, PMID: 27698303

[50] Slavin-Stewart C Phillips A Horton R. A feasibility study of home-based palliative care telemedicine in rural Nova Scotia. J Palliat Med. (2020) 23:548–51. doi: 10.1089/jpm.2019.0173, PMID: 31532325

[51] Lynch T Clark D Centeno C Rocafort J De Lima L Filbet M. Barriers to the development of palliative care in Western Europe. Palliat Med. (2010) 24:812–9. doi: 10.1177/0269216310368578, PMID: 20501511

[52] Lynch T Clark D Centeno C Rocafort J Flores LA Greenwood A. Barriers to the development of palliative care in the countries of central and Eastern Europe and the commonwealth of independent states. J Pain Symptom Manag. (2009) 37:305–15. doi: 10.1016/j.jpainsymman.2008.03.011, PMID: 19268812

[53] Bergenholtz H Missel M Timm H. Talking about death and dying in a hospital setting - a qualitative study of the wishes for end-of-life conversations from the perspective of patients and spouses. BMC Palliat Care, (2020). 19, 168. doi: 10.1186/s12904-020-00675-1 PMC7607873 33138799

[54] Kirshbaum MN Purcell B Nash S. Talking about dying and death: a focus group study to explore a local community perspective. Nurs Rep. (2011) 1:e8. doi: 10.4081/nursrep.2011.e8

[55] McIlfatrick S Hasson F McLaughlin D Johnston G Roulston A Rutherford L. Public awareness and attitudes toward palliative care in Northern Ireland. BMC Palliat Care. (2013) 12:34. doi: 10.1186/1472-684X-12-34, PMID: 24044631 PMC3848719

[56] Cox K Bird L Arthur A Kennedy S Pollock K Kumar A. Public attitudes to death and dying in the UK: a review of published literature. BMJ Support Palliat Care. (2013) 3:37–45. doi: 10.1136/bmjspcare-2012-000203, PMID: 24644327

[57] Williams SJ Seale C Boden S Lowe P Steinberg DL. Medicalization and beyond: the social construction of insomnia and snoring in the news. Health. (2008) 12:251–68. doi: 10.1177/1363459307086846, PMID: 18400832

[58] Neal A. Staff Experiences of the Media Representations of Paediatric Palliative Care: Implications for Wellbeing and career longevity. Professional Doctorate Thesis University of East London (2015).

[59] Van den Berg R Eliel MR Meijman FJ. Palliative and terminal care at home as portrayed in Dutch newspapers in 2009 compared to 2000. Eur J Gen Pract. (2011) 17:14–9. doi: 10.3109/13814788.2010.549224, PMID: 21309648

[60] Seymour J Kennedy S Arthur A Pollock P Cox K Kumar A. Public attitudes to death, dying and bereavement: A systematic synthesis. A report to the National Council for Palliative Care and National End of Life Care Programme, University of Nottingham, UK. (2009).

[61] Baker ME. Economic, political and ethnic influences on end-of-life decision-making: a decade in review. J Health Soc Policy. (2002) 14:27–39. doi: 10.1300/J045v14n03_02, PMID: 12086011

[62] Conner NE. Predictive factors of hospice use among blacks: applying Andersen's behavioral model. Am J Hosp Palliat Med. (2012) 29:368–74. doi: 10.1177/1049909111425227, PMID: 22072640

[63] Sanders C Seymour J Clarke A Gott M Welton M. Development of a peer education programme for advance end-of-life care planning. Int J Palliat Nurs. (2006) 12:216–23. doi: 10.12968/ijpn.2006.12.5.21174 16835561

[64] Enguidanos S Kogan AC Lorenz K Taylor G. Use of role model stories to overcome barriers to hospice among African Americans. J Palliat Med. (2011) 14:161–8. doi: 10.1089/jpm.2010.0380, PMID: 21265628

[65] Li L Wang F Liang Q Lin L Shui X. Nurses knowledge of palliative care: systematic review and meta-analysis. BMJ Support Palliat Care. (2023) 27:104. doi: 10.1136/spcare-2022-004104, PMID: 37369574

[66] Parajuli J Hupcey J. A systematic review on oncology nurses’ knowledge on palliative care. Cancer Nurs. (2021) 44:E311–22. doi: 10.1097/NCC.0000000000000817, PMID: 32217878

[67] Khanali-Mojen L Akbari ME Ashrafizadeh H Barasteh S Beiranvand S Eshaghian-Dorcheh A. Caregivers’ knowledge of and attitude towards palliative care in Iran. Asian Pac J Cancer Prev. (2022) 23:3743–51. doi: 10.31557/APJCP.2022.23.11.3743, PMID: 36444587 PMC9930973

